# The Early Effects on Tricuspid Annulus and Right Chambers Dimensions in Successful Tricuspid Valve Bicuspidization

**DOI:** 10.3390/jcm12124093

**Published:** 2023-06-16

**Authors:** Gintarė Bieliauskienė, Ieva Kažukauskienė, Vilius Janušauskas, Aleksejus Zorinas, Kęstutis Ručinskas, Antanas Mainelis, Diana Zakarkaitė

**Affiliations:** 1 Clinic of Cardiovascular Diseases, Institute of Clinical Medicine, Faculty of Medicine, Vilnius University, M. K. Čiurlionio 21, 03101 Vilnius, Lithuania; 2 Faculty of Mathematics and Informatics, Vilnius University, Naugarduko 24, 03225 Vilnius, Lithuania

**Keywords:** three-dimensional echocardiography, tricuspid annulus, bicuspidization, tricuspid valve quantification

## Abstract

Background: It is unclear to what degree of tricuspid annulus (TA) reduction is necessary to achieve good postoperative results in surgical bicuspidization. The study aimed to evaluate TA and right heart chamber’s dimensions before and after heart surgery; and to compare TA parameters assessed by different modalities. Methods: Forty patients underwent mitral valve surgery with or without concomitant tricuspid valve (TV) bicuspidization. Preoperative and postoperative measurements of TA dimensions were performed prospectively using two-dimensional (2D) and three-dimensional (3D) transthoracic echocardiography (TTE). Additionally, preoperative transesophageal echocardiography (TOE) was performed in the operating room prior to surgery. Results: All patients had no or mild TR immediately after surgery. There was a significant reduction in 2D and 3D parameters of the TV and right chambers in the TV bicuspidization group. However, TV leaflets’ tethering parameters did not change significantly. Preoperative 3D TTE measurements were smaller than those obtained through 3D TOE in the operation room, before surgery under general anesthesia. The 2D systolic apical 4Ch diameter and the parasternal short axis diameter mainly represent the 3D minor axis of the TA and are smaller than its 3D major axis. Conclusions: Although bicuspidization results in a one-third reduction of the TV area, tethering of the TV leaflets remains unchanged. Moreover, 3D TOE parameters of the TV under general anesthesia are larger than preoperative 3D TTE measurements. Conventional 2D measurements are insufficient for evaluating the maximum diameter of the TA.

## 1. Introduction

The prevalence of functional tricuspid regurgitation (TR) is high and increases with age [1,2]. Clinically significant functional TR is an independent predictor for mortality [3,4,5]. Irrespective of the initial etiology, functional TR is a progressive disease [6]. Therefore, optimal timing of the tricuspid valve (TV) intervention is crucial to avoid irreversible right ventricle (RV) dysfunction and to ensure good postoperative outcomes [7,8].

Surgical bicuspidization is one of the repair techniques which is replicated in transcatheter TV procedures. However, due to the absence of data on how baseline TV size impacts postoperative outcomes, the results of surgical bicuspidization are controversial. TV suture repair techniques are preferred among surgeons in patients with smaller TV annulus. However, the rigid ring repair technique is the treatment modality of choice in cases with severe annulus dilation.

Currently, no data are available on the precise reduction in tricuspid annulus (TA) size resulting from surgical procedures in humans. Surgical suture bicuspidization obliterates the posterior leaflet, resulting in bicuspidly functioning TV [9]. However, the exact degree of size reduction achieved by surgical bicuspidization and the most affected parameters of the TV is unknown. These data could be applied in catheter-based TV repair procedures.

Accurate TV sizing and extensive analysis are other challenges. Two-dimensional (2D) echocardiography is commonly used in daily practice. Unfortunately, a comprehensive TV evaluation cannot be achieved by it. Three-dimensional (3D) echocardiography offers a clearer understanding of TV assessment and provides detailed TA sizing. In addition, it enables the assessment of the spatial TV anatomy and evaluation of the grade and mechanics of TR [10]. However, there is a lack of knowledge on how 3D echocardiography parameters reflect and complement 2D measurements. Additionally, it needs to be clarified how 3D parameters assessed by different modalities (transthoracic echocardiography (TTE) and transoesophageal echocardiography (TOE)) correlate with each other.

The present study aimed to: (1) evaluate and compare TA and right heart chamber’s dimensions before and in the early stage after the heart surgery using 2D and 3D TTE, (2) compare TA measurements performed by 3D TTE and 3D TOE, and (3) compare TA measurements obtained using 2D and 3D echocardiography.

## 2. Materials and Methods

### 2.1. Study Population and Indications for Intervention

Vilnius Regional Biomedical Research Ethics Committee have granted approval for this study (protocol number 2019/6-1131-630). We prospectively carried out 3D echocardiography in 40 patients who were referred for mitral valve surgery at Vilnius University Hospital Santaros Klinikos between November 2018 and May 2022. All patients provided written consent to participate in the study. Concomitant tricuspid valve suture annuloplasty using the bicuspidization method was performed in 21 patients. The remaining 19 patients did not undergo any intervention on TV. The TV repair was performed within current guidelines [11,12]. Indications for concomitant TV repair included the presence of severe TR or mild to moderate TR with dilated TA. The TA was considered dilated when the end-diastolic diameter was ≥40 mm or >21 mm/m^2^ in 2D TTE four chambers apical view [13,14].

### 2.2. Image Acquisition

#### 2.2.1. Two-Dimensional and Three-Dimensional Transthoracic Echocardiography 

TTE was performed before and within seven days after the operation. Two-dimensional and three-dimensional TTE images were obtained using the Vivid E95 ultrasound machine (GE Healthcare; Horten, Norway) equipped with the 4Vc transducer. The study participants underwent conventional 2D TTE, including a parasternal long-axis RV inflow and parasternal short-axis at an aortic plane, the standard apical four-chamber (4Ch) view, and the apical 4Ch view optimized for the RV. Three consecutive cardiac cycles were recorded during a breath-hold with stable electrocardiographic tracing to minimize respiratory movements and obtain high-quality images suitable for TV analysis. All patients were examined in the left lateral decubitus position using grayscale second-harmonic 2D echocardiography. The image contrast, frequency depth, and sector size were adjusted to achieve an adequate frame rate for optimal visualization of the TV.

The 3D echocardiography images of the TV were acquired from the RV-focused apical view, with the gain, sector width and depth settings being adjusted prior to acquisition. The R-wave-gated acquisition was performed over four beats during a single breath-hold. Single-beat narrow volume with minimal depth was used in patients with atrial fibrillation. In patients with TR, 3D echocardiography with color Doppler was performed. All 2D and 3D echocardiography data were digitally stored and transferred to EchoPAC for further offline analysis.

#### 2.2.2. Three-Dimensional Transoesophageal Echocardiography 

TOE was performed before surgery when the patient was under general anaesthesia in the operating room. The Vivid E95 ultrasound machine (GE Healthcare; Horten, Norway) with the 6VT-D probe was used to perform the TOE. The TV was imaged at the mid-oesophageal level using a 60° to 80° imaging plane, capturing a large pyramidal dataset to encompass the entire TV. Multi-beat 3D echocardiography datasets were obtained using electrocardiographic gating, with and without color Doppler. The temporal resolution was maximized by optimizing sector width and minimizing depth to achieve an optimal volume rate. 

### 2.3. Two-Dimensional Echocardiography Measurements

The severity of TR was defined according to the guidelines of the American Society of Echocardiography and the position paper on multi-modality imaging assessment of native valvular regurgitation of the European Association of Cardiovascular Imaging (EACVI) and European Society of Cardiology (ESC) [14,15]. Grading of TR severity was performed following current guidelines with a consideration of a multiparametric approach and by using a combination of qualitative methods (TV morphology, color flow TR jet, continuous-wave (CW) signal of TR jet), semi-quantitative methods (hepatic vein flow, tricuspid inflow, proximal isovelocity surface area (PISA) radius, vena contracta (VC) width, 3D VC area) and quantitative methods (effective regurgitant orifice area (EROA), regurgitant volume (RVol)).

Left ventricular ejection fraction (LVEF) and pulmonary artery systolic pressure (PASP) were measured as recommended in guidelines [16]. The systolic TA diameter was measured in the apical four-chamber (4Ch), parasternal long axis of the right ventricular inflow tract, and parasternal short axis views in all patients. The TA end-diastolic diameter was measured in the apical 4Ch chamber view. In order to assess RV function, fractional area change (FAC) was obtained, and right ventricle (RV) size was determined by measuring the RV basal diameter, RV mid diameter, and RV length. Finally, the measurements of the right atrium (RA) were performed.

### 2.4. Three-Dimensional Images Analysis

Three-dimensional TA measurements from TTE and TOE images were gathered using a 4D Auto TVQ Tricuspid Valve Quantification software package (EchoPAC; GE Healthcare, Horten, Norway). TR mechanics are related to the anatomy of TV in mid-systole at the maximal TR velocity. Therefore, all parameters of TV were measured in mid-systole. The software provides a multiplanar reconstruction (MPR) of the TV in 4-chamber (4Ch), 2-chamber (2Ch), and short axis views. Landmarks were placed to identify the septum, free wall, anterior, posterior annulus and leaflets’ coaptation points. The cardiac cycle timing (end-diastole and end-systole) was selected and manually adjusted if necessary. The end-diastole was set as the first frame before TV closure, while the end-systole was set as the last frame before TV opening. The middle frame was selected between end-diastole and end-systole and was considered mid-systole. The segmentation process started automatically, and the TA landmarks were adjusted if needed.

The software provided various measurements of the 3D TV geometry, including TV area (the area of the non-planar surface delineated by the TA 3D contour); TV perimeter (the length of the 3D contour representing the TA circumference); 4Ch diameter (the distance between septal and lateral TA hinge points on 4Ch long axis view); 2Ch diameter (the distance between anterior and posterior TA hinge points on 2Ch long axis view); major axis (the longest diameter of the TA); minor axis (the shortest diameter of the TA); tenting volume (the volume between the leaflets and the TA surface); max tenting height (the peak distance of the valve surface to the TV plane); coaptation height (the height of the user-placed coaptation point to the 4Ch diameter of the TV annulus), and sphericity index (ratio between the minor and major axis of the TA) [17].

Reconstructed 3D echocardiography images with Color Doppler were used to measure the 3D vena contracta area [18].

### 2.5. Reproducibility Analysis

Interobserver and intraobserver variability were checked in a subset of 20 patients for the TV area and perimeter. A single observer measured these parameters from two sequentially collected images to determine intraobserver repeatability. Furthermore, the same image was analyzed by two independent observers to determine interobserver variability.

### 2.6. Surgical Procedure

All TV repairs were performed as a concomitant procedure during mitral valve surgery, using the suture bicuspidalization technique. All operations were performed in a conventional cardiac surgery fashion, under cardiopulmonary bypass, with the heart arrested. This annuloplasty technique targets the dilation of the TA that occurs in the septal to lateral and posteroseptal to anterolateral directions [19]. The procedure involves a posterior suture annuloplasty (bicuspidization) to eliminate the unsupported area of the TA and prevent its dilation.

**Figure 1:**
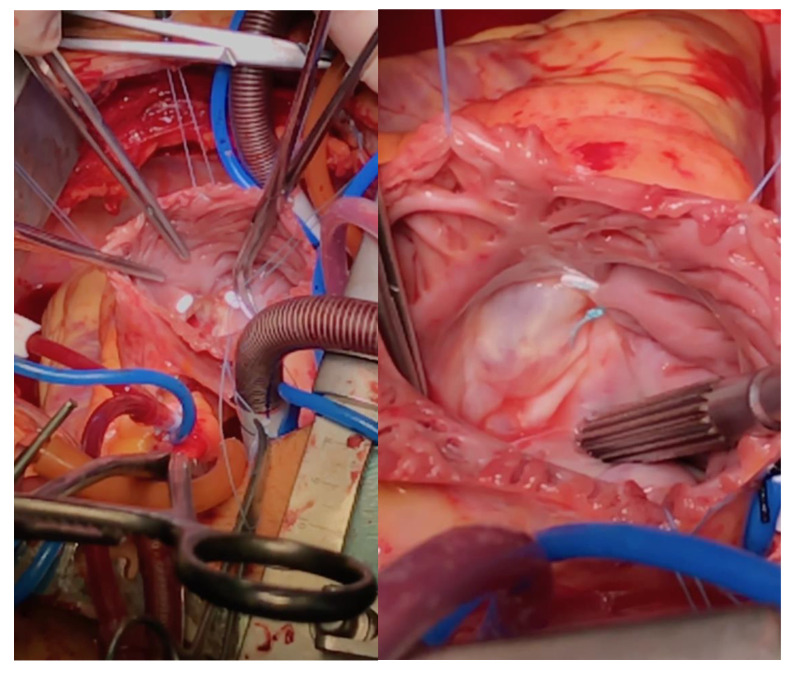
The steps of bicuspidization.

A mattress suture was placed from the anteroposterior to the posteroseptal commissure along the posterior annulus. The suture was tied to obliterate the posterior leaflet resulting in bicuspidization of the TV (Figure 1).

### 2.7. Evaluation of Early Clinical Outcomes

Early mortality was defined according to the guidelines for reporting mortality and morbidity after cardiac valve interventions as any death occurring within 30 days after surgery or during the same hospital admission. Data regarding the duration of cardiopulmonary bypass (CPB), length of stay in the intensive care unit (ICU) and hospital, and postoperative complications were collected retrospectively for all patients. Postoperative complications included new-onset atrial fibrillation, use of intra-aortic balloon pump (IABP) or extracorporeal membrane oxygenator (ECMO), resternotomy for bleeding, acute kidney injury (AKI) requiring dialysis, stroke, respiratory complications, atrioventricular block, permanent pacemaker insertion, mediastinitis, and tracheostomy.

### 2.8. Statistical Analysis

All statistical analysis was performed using R (v. 4.0.4) program package. The Shapiro–Wilk test was used to assess the normality of the quantitative variables. Normally distributed continuous variables are presented as the mean and standard deviation (SD), while other continuous variables are expressed as the median, first and third quartiles. Categorical variables are presented using frequencies and percentage.

The Independent Student’s *t*-test or nonparametric Mann–Whitney U test was used to compare quantitative variables between two independent groups, as appropriate. For comparison between two dependent quantitative dependent variables, either the paired t-test or Wilcoxon signed-rank test was used, as appropriate. Categorical variables were compared between the groups by the chi-square test or Fisher’s exact test if expected values were <5. Pearson or Spearman correlation was used to identify relations between two quantitative variables, as appropriate

To estimate inter- and intra-rater reliability, a two-way random Intraclass Correlation Coefficient (ICC) was used.

A *p*-value of less than 0.05 was considered significant.

## 3. Results

### 3.1. Characteristics of the Study Population

A total of forty patients were referred for mitral valve surgery. Baseline characteristics are listed in Table 1. The study population consisted of 26 (65%) men and 14 (35%) women, with a mean age of 61 ± 10 years. The majority of patients were symptomatic and in New York Heart Association (NYHA) functional class II (*n* = 9; 23%) or III (*n* = 29; 73%). The mean LVEF was 54.2 ± 6.6%, and the mean PASP was 44.0 ± 16.3 mmHg.

Half of the patients (*n* = 23; 58%) had atrial fibrillation. One-third of the patients (*n* = 14; 35%) had concomitant coronary artery disease. Thirty-five patients presented with organic MR, including 18 (45%) with ruptured chordae, 10 (25%) with rheumatic MV disease, six (15%) with MV prolapse, one (3%) with MV infective endocarditis, and five (45%) patients with functional MR. 

All patients were referred for mitral valve surgery, which included mitral replacement in 16 cases (40%) and mitral valve repair in 14 cases (35%). In addition, five patients had concomitant coronary artery bypass grafting, and six patients had a simultaneous aortic valve replacement.

### 3.2. Patients with Bicuspidization vs. without

Of the 40 patients who underwent mitral valve surgery, 21 had indications for concomitant TV bicuspidization; therefore, the patients were divided into two groups: with TV bicuspidization (*n* = 21); without TV bicuspidization (*n* = 19). The demographics and preoperative characteristics of the patients are summarized in Table 1**.** No significant differences were found between these two groups in terms of age, gender, body surface area (BSA), laboratory finding (hemoglobin), preoperative LVEF, PASP, NYHA functional class, the incidence of atrial fibrillation, coronary artery disease, chronic obstructive pulmonary disease, grade of preoperative TR and MR.

In the group with bicuspidization, only three patients had severe TR, while the rest of the patients had moderate, mild or no TR. There were no patients with severe TR in the group without bicuspidization. The patients in the TV bicuspidization group had significantly larger preoperative TA dimensions (TA area, perimeter, major and minor axis), 2D TA end-diastolic diameter, right ventricle (RV) basal and mid diameters, and right atrium (RA) area (Table 2). However, the effective regurgitant orifice area (EROA), regurgitant volume, fractional area change (FAC), and RV length did not differ between the groups. In addition, the two groups had no difference in preoperative MR grade.

### 3.3. Early Clinical Outcomes

Early outcomes are summarized in Table 3. There were no cases of early mortality (≤30 days) in either group. No statistically significant differences were observed between the two groups in terms of CPB time, stay in the ICU, or hospital stay. Postoperative complications, including IABP or ECMO use, resternotomy for bleeding, AKI requiring dialysis, stroke, respiratory complications, atrioventricular block, permanent pacemaker insertion, mediastinitis, and tracheostomy did not show any significant differences between the groups. However, it was noted that new-onset AF occurred more frequently in patients without bicuspidization.

### 3.4. Impact of Bicuspidization on Tricuspid Valve Dimensions

**Figure 2:**
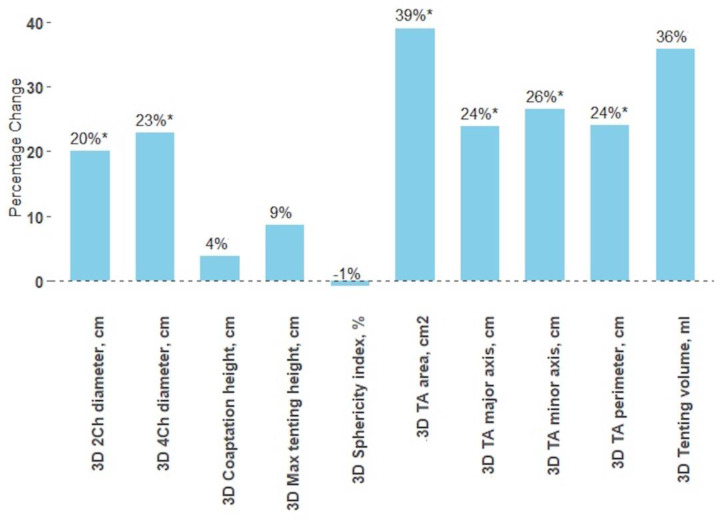
The TA parameters changes after the bicuspidization; *—*p*-value < 0.005.

There was a significant reduction in TA area (39%), perimeter (24%), major axis (24%), minor axis (26%), 4Ch diameter (23%), 2Ch diameter (20%), TA end-diastolic diameter (21%), RV basal diameter (10%) and RA area (28%) in patients who have undergone bicuspidization (Table 4). However, the max tenting height, coaptation height, tenting volume, and sphericity index did not change significantly (Figure 2).

In patients without bicuspidization, there were no significant changes in the TA geometry parameters. Additionally, this group showed a significant increase in the TA end-diastolic diameter and RV basal diameter, while the RA area significantly decreased (Table 4).

The fractional area change (FAC) increased in both groups: from 36.2 ± 11.2% to 37.0 ± 9.2% (*p* = 0.863) in bicuspidized patients and from 42.6 ± 13.4% to 45.8 ± 11.3% (*p* = 0.116) in patients without bicuspidization. However, these changes were not statistically significant.

### 3.5. Transthoracic vs. Transesophageal 3D Echocardiography

There was a significant difference between the dimensions of the TA obtained through 3D TTE before the surgery and those obtained through 3D TOE in the operation room before the surgery under general anesthesia (Table 5). The TA area, perimeter, major and minor axis were significantly smaller when measured by 3D TTE before the surgery (*p* < 0.05).

### 3.6. Comparison between 2D and 3D Transthoracic Echocardiography Measurements

The 2D and 3D echocardiographic TA measurements were performed in all patients. There was a strong correlation between 2D systolic apical 4Ch diameter and 3D minor TA axis (r = 0.645, *p* < 0.001), with no significant difference between the two measurements (3.20 ± 0.60 and 3.15 ± 0.54 cm; *p* = 0.569, respectively). Similarly, 2D parasternal short axis diameter had a high correlation with 3D TA minor axis (r = 0.628, *p* < 0.001), and there was no significant difference between these two measurements (3.22 ± 0.61 and 3.15 ± 0.54 cm *p* = 0.439, respectively).

**Figure 3:**
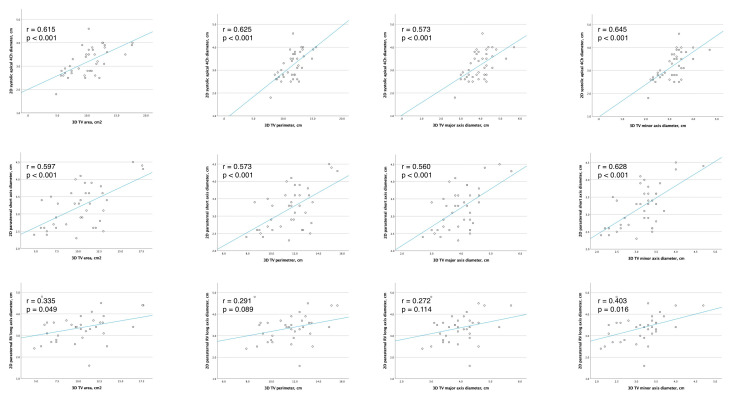
Correlation between 2D and 3D echocardiographic TA measurements.

**Figure 4:**
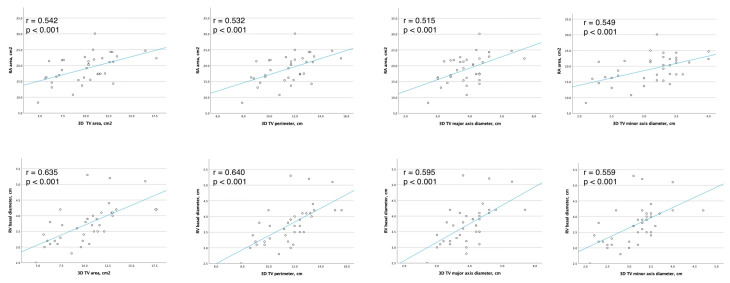
Correlation between RA area, RV basal diameter and 3D TA measurements.

The 3D TA major axis, which represents the largest diameter of an ellipse that fits the TA shape, was significantly larger than 2D systolic apical 4Ch diameter (3.95 ± 0.62 cm vs. 3.20 ± 0.60 cm, *p* < 0.001, respectively). Other correlations are presented in Figure 3 and Figure 4.

### 3.7. Interobserver and Intraobserver Variability in Annular Measurements

The interobserver and intraobserver variability of 3D echocardiographic measurements was assessed and found to have high levels of consistency. The intraclass correlation coefficients (ICC) for the TA area were 0.98 (*p* < 0.001) and 0.98 (*p* < 0.001), respectively. The ICC values for the TA perimeter were 0.97 (*p* < 0.001) and 0.96 (*p* < 0.001) for interobserver and intraobserver variability, respectively. The ICC values indicated near-perfect consistency for both TA area and perimeter measurements. However, the measurement of the TA area showed slightly higher consistency than that of the TA perimeter.

## 4. Discussion

To our best knowledge, this is the first study to evaluate the detailed 3D TA changes in the early stage after surgical treatment. The current study describes the changes in the dimensions of the TV before and after successful bicuspidization using 3D echocardiography. Additionally, the study compares the 3D TTE and TOE measurements of the TV and the TA’s 2D and 3D echocardiographic metrics.

The main findings of this study can be summarized as follows: (1) surgical bicuspidization significantly decreased TA dimensions, with a one-third reduction in TA area and a one-quarter reduction in perimeter; however, TV leaflets tethering remained unchanged; (2) 3D TOE TA measurements performed in the operating room before surgery under general anesthesia were significantly larger than preoperative 3D TTE TA parameters; (3) the 2D systolic apical 4Ch diameter and parasternal short axis diameter primarily corresponded to the 3D minor axis and were significantly smaller than the 3D major axis of the TA.

According to current guidelines, the indications for TV intervention include severe TR and mild or moderate TR with a dilated TA [11]. In our study, only three of all bicuspidized patients had severe TR, while the remaining patients had moderate or less TR. Therefore, the decision to perform TV repair was mainly based on the TA dimensions. The TA end-diastolic diameter can be easily measured during routinely performed 2D TTE, and it is expected that 2D measurements reflect the degree of TA enlargement. However, our study demonstrated that 2D parameters, such as systolic apical 4Ch diameter and parasternal short axis diameter, are significantly smaller than the 3D major axis and correspond to the 3D minor axis. This conclusion is supported by some studies that have reported that the TA diameter measured in the 4-chamber view by 2D echocardiography underestimates the 4Ch diameter obtained by 3D echocardiography [20,21,22]. Thus, solely 2D measurements may not provide an accurate assessment of the degree of TA dilation, leading to a potential delay in the appropriate timing of TV intervention. The proper timing of TV intervention should be determined after a comprehensive TV, RV and RA assessment [23]. J. Dreyfus et al., showed that TV intervention is often performed too late [7]. An earlier TV surgery may improve immediate and mid-term outcomes [7,24,25]. I. Kara et al., found in their meta-analysis that a more aggressive strategy may be considered during the left-sided valve surgery in patients with mild to moderate functional TR at baseline to prevent progression of TR [26]. A recent study by A. Piperata et al., showed that mitral valve surgery combined with simultaneous TV suture bicuspidization, as a preventive measure for mild to moderate TR, significantly reduces the long-term progression of TV regurgitation compared to individuals undergoing mitral valve surgery alone. Additionally, this technique demonstrated similar survival rates at 30-day and in the long-term, when compared to isolated mitral valve surgery [27]. 

In our study, the selected patients underwent left-heart surgery and concomitant TV repair using the suture annuloplasty by the bicuspidization method. In their study, Ravi K. Ghanta et al., revealed that both bicuspidization and ring annuloplasty were equally effective and durable in reducing TR up to 3 years postoperatively [28]. However, S. Hirji et al., reported that bicuspidization annuloplasty had a higher incidence of late clinically significant TR recurrence and lower freedom from TV reoperation compared to the ring annuloplasty technique [29]. Neither study reported how TA size influenced the choice of annuloplasty technique. We hypothesise that the size of the TA is an essential factor in selecting the annuloplasty modality. Praz F. et al., suggested that excessive annular dilatation is unsuitable for transcatheter annuloplasty procedures [30]. Further studies on long-term outcomes are needed to evaluate the impact of TA size on different repair techniques. It is likely that for significantly enlarged TA with severe TR, suture annuloplasty may be insufficient, and ring annuloplasty may be necessary for better long-term results.

Our study showed a reduction in all TV annulus parameters, including TA area, perimeter, major and minor axis, 4Ch diameter, 2Ch diameter, TA end-diastolic diameter, RV basal diameter, and RA area after the bicuspidization. Specifically, surgical bicuspidization achieved a one-third reduction in TA area and a one-quarter reduction in perimeter, major and minor axis. These findings align with the data from a recently published larger cohort study by A. Piperata, which showed a 30% reduction in TV annulus diameter after bicuspidization postoperatively. Moreover, this reduction was maintained during the latest follow-up [27].

So far, little information is available regarding the effect of bicuspidisation on the precise reduction of TA size. There is a scarcity of data on how bicuspidisation affects animal hearts ex vivo. F. Sulejmani el al., studied the mechanics of the percutaneous TV bicuspidization in six porcine hearts using a novel device. The investigators found that after bicuspidization, the TA became more circular. Compared to the percutaneous bicuspidization procedure using the Trialign^TM^ device (Mitralign, Inc., Tewksburry, MA, USA) in humans, percutaneous bicuspidization in the porcine model showed a more significant reduction in the TV annular area than the human results [31].

M. Mathur et al., investigated the effect of TV annular downsizing using DeVega suture annuloplasty in ten anesthetized sheep. DeVega suture annuloplasty resulted in TA shape and metrics changes, including a reduction in the area, perimeter, global height, and eccentricity. The reduction in TV annular area was primarily driven by the compression of the anterior annulus [32].

Currently, there is limited data available regarding the precise reduction in TA size resulting from surgical bicuspidization in humans. With the increasing number of transcatheter procedures, improving our knowledge of TA changes during surgical suture annuloplasty is important. Trialign^TM^ (Mitralign, Inc., Tewksburry, MA, USA) is an annuloplasty technique, which mimics Kay’s procedure by cinching the posterior portion of the tricuspid annulus. The SCOUT trial reported a reduction of the TA area from 11.6 (8.5, 17.4) cm^2^ to 10.0 (8.1, 15.2) cm^2^ using the Trialign^TM^ device [33]. Similarly, the Cardioband^TM^ (Edwards Lifesciences, Irvine, CA, USA) tricuspid system reduced the annulus from 14.3 ± 2.7 cm^2^ to 10.0 ± 2.4 cm^2^ [34]. However, surgical bicuspidization achieved a greater reduction in TV area (39%) compared to transcatheter techniques, including Trialing^TM^ device (14%) or Cardioband^TM^ system (30%). Therefore, understanding the exact degree of TA size reduction required for successful bicuspidization can be applied to percutaneous annuloplasty procedures.

However, our study found that bicuspidization did not significantly change the TV leaflets tethering parameters in the early period after the surgery. Severe TV leaflets tethering might be another reason why annuloplasty might be insufficient to correct TR. Fukuda S. et al., identified that the tethering of TV is an independent predictor of residual TR early after TV annuloplasty [35]. Although the bicuspidization reduces the TA area and dimensions, it has little effect on the TV leaflets tethering. Therefore, TV size and leaflets tethering should be considered when choosing a TV repair technique.

Regarding early postoperative outcomes, our results revealed that bicuspidization did not increase mortality, CPB time, length of stay in the ICU, hospital stay, or the risk of postoperative complications. The study by A. Piperata et al., supports our findings. Although they reported a longer CPB time in the bicuspidised patient group, this technique did not increase perioperative mortality and complications [27].

After cardiac surgery, the RV FAC remained unchanged in our patient cohort. Although there was a slight increase in FAC in both groups, these changes were not statistically significant. Our findings are consistent with several other studies that have examined the impact of cardiothoracic surgery on the right ventricle (RV). After cardiac surgery, there are fundamental changes in the contractile pattern of the RV. Measures of longitudinal RV shortening, such as TAPSE or the systolic tricuspid annular velocity, typically decrease postoperatively. In contrast, RV ejection fraction and RV FAC appear to remain stable or even increase after cardiac surgery [36,37,38,39]. It could be explained by deformation analysis of RV. M. Donauer et al., demonstrated that RV circumferential and radial contraction do not show significant changes after cardiac surgery. However, there is a significant decrease in peak longitudinal systolic strain of the RV lateral wall [40]. RV FAC, which encompasses both longitudinal and transverse contraction of the RV lateral free wall, remains preserved post-surgery [37,40].

The TV could be visualized on either transthoracic or transoesophageal echocardiography. However, the measurements achieved by both modalities vary. We found that the dimensions of the TA obtained by 3D TTE were smaller than those obtained by 3D TOE. We hypothesize that the discrepancy in measurements may be attributed to the timing of the study. The 3D TTE was performed before surgery, while the 3D TTE was performed in the operating room just before the surgery with the patient under general anaesthesia. There is limited data available on the impact of general anesthesia on the dimensions, dynamics, and geometry of the TV. T. Jaswiec at el. showed in their animal studies using sonomicrometry [41] that general anesthesia and mechanical ventilation reduced TA dynamics and RV contractility, but the annular and subvalvular geometry remained unchanged. As reported in their study, the TA area and perimeter contraction decreased with anesthesia, primarily due to reduced septal contraction. Furthermore, the end-systolic and end-diastolic TA area were larger in anesthetized patients, although not significantly [41]. Previous studies on the mitral valve have demonstrated that intraoperative transesophageal echocardiography performed under general anesthesia may significantly underestimate the severity of functional mitral regurgitation [42,43,44]. Similar mechanisms may be expected to influence the severity of TR under general anesthesia and mechanical ventilation.

Since the TA is a spatial structure, only 3D echocardiography provides a better understanding of the TV geometry, and helps to assess TV dimensions more accurately. In addition, 2D echocardiography should be used only in combination with 3D echocardiography for comprehensive TV evaluation. It confirms the need and importance of establishing normal 3D TA values for future guidelines.

There were several limitations in this study. Firstly, it was a single-center study with a small sample size. Secondly, the analysis did not involve the relationships between TV dimensions and both RA and RV volumes. Thirdly, the analysis was limited to short-term outcomes and further long-term follow-up results is necessary.

## 5. Conclusions

Bicuspidization significantly reduces TA area and dimensions but minimally impacts the leaflets tethering.

In patients under general anesthesia, 3D TEE parameters of the TV are larger than in conscience patients’ 3D TTE measurements.

The 2D systolic measurements of the apical 4Ch diameter and the parasternal short axis diameter are insufficient for assessing the maximum dimensions of the TA.

## Figures and Tables

**Table 1:** Patients’ demographics and preoperative characteristics overall and by groups.

	Overall, *n* = 40	With Bicuspidization, *n* = 21	Without Bicuspidization, *n* = 19	*p*-Value
Age, yrs	60.9 ± 9.5	62.7 ± 9.4	59.0 ± 9.5	0.221
Female/male	14 (35%)/26 (65%)	7 (33%)/14 (67%)	7 (37%)/12 (63%)	0.816
Weight, kg	78.6 ± 18.9	77.7 ± 18.5	79.5 ± 19.8	0.818
Height, cm	171.3 ± 9.6	171.2 ± 9.8	171.4 ± 9.6	0.914
BSA, m^2^	1.92 ± 0.27	1.91 ± 0.27	1.93 ± 0.28	0.819
Hemoglobin, g/L	140.6 ± 14.7	140.7 ± 14.6	140.5 ± 15.3	0.972
GFR, mL/min	75.8 ± 18.8	67.3 ± 17.9	85.1 ± 15.3	0.005
BNP, ng/L	225.2 (76.1–526.2)	411.7 (210.6–855.0)	82.0 (49.1–225.2)	0.003
Atrial fibrillation	23 (58%)	14 (67%)	9 (47%)	0.218
Coronary artery disease	14 (35%)	8 (38%)	6 (31%)	0.666
COPD	2 (5%)	2 (9%)	0 (0%)	0.168
NYHA class:				0.004
I	2 (5%)	0 (0%)	2 (11%)	
II	9 (23%)	1 (5%)	8 (42%)	
III	29 (73%)	20 (95%)	9 (47%)	
Preoperative LVEF, %	54.2 ± 6.6	54.5 ± 5.0	53.8 ± 8.2	0.510
Preoperative PASP, mmHg	44.0 ± 16.3	44.1 ± 16.1	43.8 ± 17.3	0.877
Preoperative TR grade:				0.074
No TR	9 (23%)	3 (14%)	6 (32%)	
Mild	19 (48%)	8 (38%)	11 (58%)	
Moderate	9 (23%)	7 (33%)	2 (10%)	
Severe	3 (8%)	3 (14%)	0 (0%)	
Preoperative MR grade:				0.793
Mild	3 (8%)	1 (5%)	2 (10%)	
Moderate	1 (3%)	1 (5%)	0 (0%)	
Severe	36 (90%)	19 (90%)	17 (90%)	
Left heart disease:				0.551
Functional MR	5 (12%)	2 (10%)	3 (16%)	
MV flail	18 (45%)	9 (42%)	9 (47%)	
MV prolapse	6 (15%)	2 (10%)	4 (21%)	
Rheumatic MS, MR	10 (25%)	7 (33%)	3 (16%)	
Infective endocarditis	1 (3%)	1 (5%)	0 (0%)	
MV surgery type:				0.796
MV prosthesis	16 (40%)	8 (38%)	8 (42%)	
MV repair	24 (60%)	13 (62%)	11 (58%)	
Concomitant surgery:				0.178
CABG	5 (13%)	3 (14%)	2 (11%)	
Aortic valve prosthesis	6 (15%)	1 (5%)	5 (26%)	

Values are mean ± SD, *n* (%), or median (interquartile range). BNP—brain natriuretic peptide; BSA—body surface area; CABG—coronary artery bypass graft; COPD—chronic obstructive pulmonary disease; GRF—glomerular filtration rate; LVEF—left ventricular ejection fraction; MR—mitral regurgitation; MS—mitral stenosis; MV—mitral valve; NYHA—New York Heart Association; PASP—pulmonary artery systolic pressure; TR—tricuspid regurgitation.

**Table 2:** Preoperative TTE findings of patients with bicuspidization and without bicuspidization.

	With Bicuspidization, *n* = 21	Without Bicuspidization, *n* = 19	*p*-Value
3DE parameters			
TA area, cm^2^	11.8 ± 3.0	8.6 ± 2,5	0.001
TA perimeter, cm	12.5 ± 1.6	10,6 ± 1,6	0.001
TA major axis, cm	4.2 ± 0.6	3.7 ± 0.5	0.002
TA minor axis, cm	3.4 ± 0.5	2.8 ± 0.5	0.001
4Ch diameter, cm	3.5 ± 0.6	3.3 ± 0.6	0.257
2Ch diameter, cm	3.5 ± 0.4	3.1 ± 0.5	0.040
Max tenting height, cm	0.58 ± 0.17	0.53 ± 0.19	0.439
Coaptation height, cm	0.52 ± 0.33	0.41 ± 0.21	0.272
Tenting volume, mL	1.4 (1.0–2.1)	1.1 (0.8–1.2)	0.095
Sphericity index, %	82.9 ± 9.5	77. 7 ± 7.4	0.109
2DE parameters			
Systolic apical 4Ch diameter, cm	3.4 ± 0.6	3.0 ± 0.5	0.013
Systolic parasternal long axis diameter, cm	3.4 ± 0.7	3.3 ± 0.6	0.447
Systolic parasternal short axis diameter, cm	3.3 ± 0.6	3.1 ± 0.6	0.271
2D TA end-diastolic	3.9 ± 0.5	3.3 ± 0.5	0.002
diameter, cm			
RV basal diameter, cm	4.0 ± 0.5	3.5 ± 0.6	0.004
RV mid diameter, cm	3.2 ± 0.5	2.8 ± 0.6	0.006
RV length, cm	7.1 ± 0.9	7.1 ± 0.8	0.878
FAC, %	36.2 ± 11.2	42.6 ± 13.4	0.113
RA area, cm^2^	21.5 ± 3.5	17.0 ± 4.2	0.001
EROA, cm^2^	0.25 (0.07–0.34)	0.13 (0.12–0.36)	0.982
Regurgitant volume, mL	21.0 (7.0–26.0)	11.5 (8.3–20.3)	0.387
TR grade:			0.074
No TR	3 (14%)	6 (32%)	
Mild	8 (38%)	11 (58%)	
Moderate	7 (33%)	2 (10%)	
Severe	3 (14%)	0 (0%)	
MR grade:			0.793
No MR	0 (0%)	0 (0%)	
Mild	1 (5%)	2 (10%)	
Moderate	1 (5%)	0 (0%)	
Severe	19 (90%)	17 (90%)	

Values are mean ± SD, *n* (%), or median (interquartile range). EROA—effective regurgitant orifice area; FAC—fractional area change; 4Ch—four chambers; MR—mitral regurgitation; RA—right atrium; RV—right ventricle; 3DE—three-dimensional echocardiography; TA—tricuspid annulus; TR—tricuspid regurgitation; 2Ch—two chambers; 2DE—two-dimensional echocardiography.

**Table 3:** Early clinical outcomes in both groups.

	With Bicuspidization, *n* = 21	Without Bicuspidization, *n* = 19	*p*-Value
CPB time	156 ± 39	144 ± 49	0.383
Early mortality (≤30 days)	0 (0%)	0 (0%)	-
ICU stay (days)	4 (3; 6)	3 (2; 4)	0.488
Hospital stay (days)	17 (13; 28)	16 (12; 21)	0.491
Postoperative complications:			
New-onset AF	2 (9.5%)	7 (36.8%)	0.039
IABP or ECMO	1 (4.8%)	0 (0%)	0.335
Resternotomy for bleeding	1 (4.8%)	2 (10.5%)	0.489
AKI requiring dialysis	1 (4.8%)	1 (5.3%)	0.942
Stroke	1 (4.8%)	2 (10.5%)	0.489
Respiratory complication	4 (19.0%)	2 (10.5%)	0.451
Atrioventricular block	3 (14.3%)	3 (15.8%)	0.894
Permanent pacemaker insertion	2 (9.5%)	3 (15.8%)	0.550
Mediastinitis	2 (9.5%)	0 (0%)	0.168
Tracheostomy	1 (4.8%)	0 (0%)	0.335

Values are mean ± SD, *n* (%), or median (interquartile range). AF—atrial fibrillation; AKI—acute kidney injury; CPB—cardiopulmonary bypass; ECMO—extracorporeal membrane oxygenator; IABP—intra-aortic balloon pump; ICU—intensive care unit.

**Table 4:** Preoperative and postoperative TTE findings in two groups.

KERRYPNX	With Bicuspidization, *n* = 21			Without Bicuspidization, *n* = 19		
	Before	After	*p*-Value	Before	After	*p*-Value
3DE parameters						
TA area, cm^2^	11.8 ± 3.0	7.2 ± 3.3	<0.001	8.6 ± 2,5	9.1 ± 2.2	0.346
TA perimeter, cm	12.5 ± 1.6	9.5 ± 2.1	<0.001	10.6 ± 1,6	10.9 ± 1.4	0.449
TA major axis, cm	4.2 ± 0.6	3.2 ± 0.7	<0.001	3.7 ± 0.5	3.7 ± 0.4	0.544
TA minor axis, cm	3.4 ± 0.5	2.5 ± 0.6	<0.001	2.8 ± 0.5	3.0 ± 0.5	0.172
4Ch diameter, cm	3.5 ± 0.6	2.7 ± 0.6	0.010	3.3 ± 0.6	3.3 ± 0.5	0.780
2Ch diameter, cm	3.5 ± 0.4	2.8 ± 0.5	0.049	3.1 ± 0.5	3.1 ± 0.6	0.796
Max tenting height, cm	0.60 (0.40–0.70)	0.50 (0.40–0.65)	0.751	0.53 ± 0.19	0.48 ± 0.15	0.763
Coaptation height, cm	0.40 (0.33–0.60)	0.50 (0.40–0.60)	0.487	0.41 ± 0.21	0.31 ± 0.20	0.262
Tenting volume, mL	1.4 (1.0–2.1)	0.9 (0.5–1.3)	0.068	1.1 (0.8–1.2)	0.8 (0.4–1.7)	0.962
Sphericity index, %	82.9 ± 9.5	83.6 ± 5.7	0.632	77.7 ± 7.4	78.8 ± 8.0	0.207
2DE parameters						
2D TA end-diastolic diameter, cm	3.9 ± 0.5	3.1 ± 0.6	<0.001	3.3 ± 0.5	3.7 ± 0.5	<0.001
RV basal diameter, cm	4.0 ± 0.5	3.6 ± 0.6	0.020	3.5 ± 0.6	3.9 ± 0.6	0.001
RV mid diameter, cm	3.2 ± 0.5	3.0 ± 0.6	0.108	2.8 ± 0.6	3.1 ± 0.6	0.052
RV length, cm	7.1 ± 0.9	7.3 ± 0.7	0.228	7.1 ± 0.8	7.1 ± 0.9	0.956
FAC, %	36.2 ± 11.2	37 ± 9.2	0.863	42.6 ± 13.4	45.8 ± 11.3	0.116
RA area, cm^2^	21.5 ± 3.5	15.5 ± 5.1	<0.001	17.0 ± 4.2	14.7 ± 4.5	0.001
EROA, cm^2^	0.25 (0.07–0.34)	0.08 (0.08–0.09)	0.043	0.13 (0.12–0.36)	0.15 (0.10–0.19)	1.000
Regurgitant volume, mL	21.0 (7.0–26.0)	5.0 (4.0–5.0)	0.041	11.5 (8.3–20.3)	11.0 (6.3–13.3)	0.364
TR grade:			<0.001			0.21
No TR	3 (14%)	13 (62%)		6 (32%)	10 (53%)	
Mild	8 (38%)	8 (38%)		11 (58%)	9 (47%)	
Moderate	7 (33%)	0 (0%)		2 (10%)	0 (0%)	
Severe	3 (14%)	0 (0%)		0 (0%)	0 (0%)	
MR grade:			<0.001			<0.001
No MR	0 (0%)	12 (57%)		0 (0%)	16 (84%)	
Mild	1 (5%)	6 (29%)		2 (10%)	2 (11%)	
Moderate	1 (5%)	3 (14%)		0 (5%)	1 (5%)	
Severe	19 (90%)	0 (0%)		17 (90%)	0 (0%)	

Values are mean ± SD; median (interquartile range) or *n* (%); EROA—effective regurgitant orifice area; FAC—fractional area change; 4Ch—four chambers; MR—mitral regurgitation; RA—right atrium; RV—right ventricle; 3DE—three-dimensional echocardiography; TA—tricuspid annulus; TR—tricuspid regurgitation; 2Ch—two chambers; 2DE—two-dimensional echocardiography.

**Table 5:** Transthoracic and transesophageal 3DE findings in all patients.

	Transthoracic 3DE	Transesophageal 3DE	*p*-Value
TA area, cm^2^	9.9 ± 3.3	11.4 ± 2,5	0.002
TA perimeter, cm	11.4 ± 1.9	12.4 ± 2.0	0.002
TA major axis, cm	3.9 ± 0.6	4.1 ± 0.5	0.016
TA minor axis, cm	3.1 ± 0.6	3.4 ± 0.5	0.004

Values are mean ± SD. TA—tricuspid annulus; 3DE—three-dimensional echocardiography.

## Data Availability

The data presented in this study are available on request from the corresponding author, GB.
